# Pediatric Methicillin-Resistant *Staphylococcus aureus* Osteoarticular Infections

**DOI:** 10.3390/microorganisms6020040

**Published:** 2018-05-04

**Authors:** Ashlesha Kaushik, Helen Kest

**Affiliations:** 1 Pediatric Infectious Disease, Unity Point Health and Siouxland Medical Education Foundation, 2720 Stone Park Blvd, Sioux City, IA 51104, USA; 2 Pediatric Infectious Disease, Department of Pediatrics St. Joseph’s Children’s Hospital 703, Main Street, Paterson, NJ 07503, USA; KestH@sjhmc.org

**Keywords:** osteoarticular infections, methicillin-resistant *Staphylococcus aureus*, children

## Abstract

Osteoarticular infections (OSI) are a significant cause of hospitalizations and morbidity in young children. The pediatric patient with OSI presents unique challenges in diagnosis and management due to higher morbidity, effect on growth plate with associated long-lasting sequelae, and challenges in early identification and management. Methicillin-resistant *Staphylococcus aureus* (MRSA), first described in the 1960s, has evolved rapidly to emerge as a predominant cause of OSI in children, and therefore empiric treatment for OSI should include an antibiotic effective against MRSA. Characterizing MRSA strains can be done by antimicrobial susceptibility testing, detection of Panton–Valentine leukocidin (PVL) gene, staphylococcal cassette chromosome mec (SCCmec) typing, pulsed-field gel electrophoresis (PFGE), and multilocus sequence typing (MLST). Worldwide, community-onset methicillin-resistant staphylococcal disease is widespread and is mainly associated with a PVL-producing clone, ST8/USA300. Many studies have implied a correlation between PVL genes and more severe infection. We review MRSA OSI along with the pertinent aspects of its pathogenesis, clinical spectrum, diagnosis, and current guidelines for management.

## 1. Introduction

*Staphylococcus aureus* (*S. aureus*) is a major cause of bacterial infections in humans worldwide. Methicillin-resistant *Staphylococcus aureus* (MRSA) is a leading cause of bacteremia and invasive diseases that include pulmonary, skin, and soft tissues and musculoskeletal infections in children [1,2].

The first outbreaks of MRSA-related infections were first reported in European hospitals in the early 1960s [3]. This led to the classification of *S. aureus* as health-care-associated (HA) and community-associated (CA) infections based on risk factors, place of acquisition, and genetic or antibiotic profiles. Over the years, strains of MRSA previously characterized as CA infections have been increasingly described as HA infections among hospitalized patients [4]. In the U.S., virulent community-associated MRSA (CA-MRSA) clones, characterized by the presence of the cytoxin Panton–Valentine leukocidin (PVL), have become increasingly common over the past several decades, spreading first in the community and later in healthcare facilities. This distinction between CA-MRSA and HA-MRSA is beginning to fade.

Infections caused by MRSA have become endemic in several developed countries and have reached epidemic proportions globally. MRSA disease outbreaks are increasing in many countries and over the past 2–3 decades, PVL-producing MRSA strains have been described in every continent [5].

MRSA has been a clinically significant pathogen in musculoskeletal infections for several decades. Currently, *S. aureus* is the leading cause of skin/skin structure infections and osteoarticular infections (OSI) at most children’s hospitals, and MRSA has emerged as a common pathogen with more soft-tissue destruction, a rapid spread, and a higher mortality rate [6]. Consequently, clinicians and hospital epidemiologists have become more vigilant in creating and implementing policies that address screenings, empiric antibiotic treatment, and prevention of complications.

OSI is a significant cause of morbidity worldwide in young children due to its potentially serious sequelae, especially on the growth plate. OSI can manifest as osteomyelitis, septic arthritis, or an osteomyelitis with adjacent septic arthritisor spondylodiscitis. OSI can be divided into three types according to the source of infection: hematogenous (from blood); secondary (spread from a contiguous site of infection); or secondary to direct inoculation.

Osteomyelitis can be limited to a single portion of a bone or can involve marrow, cortex, periosteum, and the surrounding soft tissue. Osteomyelitis in children usually results from hematogenous spread, which is due to seeding of bacteria following minor trauma to the bone metaphysis where blood flow is rich but sluggish. The most commonly affected sites are the long bones of the femur and tibia [7]. Infection can spread to the adjacent joint space in sites (e.g., proximal ends of the femur, humerus, and tibia) where the metaphysis is intracapsular.

In young children less than 18 months, hematogenous spread occurs from the metaphysis to the epiphysis through the transphyseal vessels to the adjoining joint space [7].

In older children, infection is usually contained by the growth plate and the joint is spared unless the metaphysis is intracapsular.

## 2. Pathophysiology

### 2.1. Characteristics of S. aureus

*S. aureus* is a commensal bacterium whose primary reservoir is the nares but is also found in other areas, such as the axillae, vagina, pharynx, and/or damaged skin surfaces [8]. Disease typically follows a breach of the skin or mucosal barrier through which *S.aureus* gains access to adjoining and/or deeper tissues or the bloodstream. Various virulence factors produced by *S. aureus*, including PVL and many other factors, such as members of the bi-component leukocidins (LukED, HlgACB, and LukAB/GH), facilitate its colonization, adaptation, and its remarkable capacity to cause diseases of varying severity from minor skin infections to life-threatening infections [9]. The high virulence of *S. aureus* is expressed in its unique ability to cause disease in previously normal tissue at all sites [10]. 

*S. aureus* Clonal Complexes may be characterized by:

1. Multilocus sequence typing (MLST), a molecular technique based on *S. aureus* genotypic sequencing of specific housekeeping genes, which are genes typically expressed in all *S. aureus* cell types. The nucleotide variations or allele of seven housekeeping genes provide a discriminatory allelic profile, known as a sequence type (ST), for each *S. aureus* isolate. Classification into unique clonal complexes (CC) is based on isolates with identical (≥5 or more) genes [11,12]. The very first MRSA clinical isolates described were ST250 and members of CC8.
2. Pulsed-field gel electrophoresis (PFGE) uses electrophoresis to separate *S. aureus* genomic fragments according to size. Related strains are clustered based on a similarity coefficient [13]. The Centers for Disease Control and Prevention (CDC) national PFGE database for *S. aureus* uses the “USA” designation; an example is the USA300, a PVL-positive MRSA and the most common cause of community-acquired skin and soft-tissue infections.
3. *Spa* typing [11], a sequence-based analysis of variable number tandem repeats in the gene encoding protein A (*Spa*). It is a relatively inexpensive method of determining epidemiological relationships.
4. Genomic sequencing and analysis of *S. aureus* population provides insight into *S aureus* epidemiology, adaptability, virulence and resistance patterns. Lineages of *S. aureus* populations show genetic variability as well as inter-genetic differences. Ten dominant lineages cause colonization and infection in humans. Genomic sequencing has led to new tools enabling construction of genetically modified clinical *S. aureus* isolates. For example, genomic analysis of the clinical vancomycin-resistant *S. aureus* (VRSA) isolates identifies the genetic basis of resistance to multiple antibiotics and suggests multiple strains with variable sensitivity patterns can affect the same patient [14].

*S. aureus* methicillin resistance is due to an acquired penicillin-binding protein, PBP2a. The mecA gene, coding for this 78-kDa PBP2a protein, causes intrinsic resistance to methicillin and other β-lactam antibiotics. In MRSA, foreign PBP2a prevents binding of β-lactam antibiotics, resulting in broad beta-lactam antibiotic class resistance (penicillins, cephalosporins, and carbapenems).

The mecA gene is located on a mobile genomic island, also known as staphylococcal cassette chromosome mec (SCCmec) [15]; resistance levels range from phenotypically susceptible to highly resistant depending on the MRSA strain’s genetic background.

Eleven SCCmec (type I to XI) types are recognized. Due to additionally integrated genes, some SCCmec types cause resistance to multiple antibiotics; for example, in type II and III, integrated plasmid pUB110 harbors the ant (4′) gene that encodes resistance to multiple aminoglycosides. CA-MRSA harbors type IV, V, or VII and is often associated with the presence of the PVL toxin.

### 2.2. Osteomyelitis

*S. aureus* cell-surface receptors, known as MSCRAMMs (microbial surface components recognizing adhesive matrix molecules), mediate adherence to components of bone matrix. [16]. Once attached to bone, *S. aureus* demonstrates the ability to establish intracellular persistence due to its metabolically quiescent, persisting phenotype [17]; this phenomenon may explain the need for more prolonged courses of antibiotics in bone infections.

Following invasion of bone tissue, an inflammatory response produces increased vascular leak and edema which leads to increased bone pressure, decreased pH, and oxygen tension. These create a circle of medullary circulation compromise that favors the spread of infection. Additional tissue lysis occurs as toxic radicals and proteolytic enzymes are generated as phagocytes attempt to contain invading *S. aureus*. Microorganisms, neutrophils, and thrombosed blood vessels are therefore the principal histologic findings in acute osteomyelitis. Ischemic necrosis of bone leads to sequestra formation, which is made of up of devascularized fragments.

Bone remodeling and healing requires the coordinated interplay of tissue resorption by osteoclasts and bone formation by osteoblasts [7].

### 2.3. Septic Arthritis

*S. aureus* has a high degree of selectivity for the synovium, which is probably related to its virulence properties that include adherence and toxin production. Septic arthritis mostly results through hematogenous *S. aureus* seeding of the vascular synovial lining. Direct implantation through trauma is the second-most common way *S. aureus* gains access to the joint space. Rarely, septic arthritis occurs as a secondary process following penetrating injuries (such as bites) or after trauma to the joint despite absence of obvious skin breaks.

The pathogenesis of acute septic arthritis is more complex and is driven by the interaction between *S. aureus* and the resultant host immune response.

Seeding of *S. aureus* into the joint space elicits production of proinflammatory cytokines (such as IL-1β, IL-6, and TNF-α) and C-reactive protein due to the response of the innate immune system to the presence of the peptidoglycan wall (via *N*-formylmethionine proteins and teichoic acids). Bacterial DNA (specifically unmethylated CpG motifs) also elicits an intense inflammatory response [10,16]. Experimental models show that after an intra-articular injection of 10^5^ *S. aureus* organisms, *Neisseria gonorrhoeae*, or *Staphylococcus epidermidis* into the knee joint, resultant significant joint destruction occurred only with *S. aureus* [18].

## 3. Clinical Spectrum of MRSA OSI

*S. aureus* causes most of the OSI in children and 50% or more of the invasive isolates are CA-MRSA [6]. The manifestations of MRSA OSI in children include osteomyelitis, septic arthritis, a combination of both (i.e., osteomyelitis with adjacent septic arthritis), or, rarely, spondylodiscitis. Approximately 15–50% of pediatric OSI are a combination involving both the joint and the bone and tend to be more serious, with higher levels of inflammatory markers, more sequelae, and often requiring a longer duration of antibiotic therapy [19,20,21,22]. 

Most pediatric OSI involve lower extremities and the large weight bearing joints. The most common sites for osteomyelitis are the lower leg bones (35%) and the femur (27%) followed by the pelvis (15%) and feet bones (11%), while the most common sites for septic arthritis are the knee joint (40%), the hip (30%), and the ankle (23%) [6]. The characteristic presenting features are fever and pain in the extremity with an inability to bear weight; however, younger children are often not able to localize the pain. While osteomyelitis may have subtle objective signs, such as point tenderness and minimal redness, septic arthritis can present with joint swelling, erythema and tenderness [23]. A preceding history of trauma/injury prior to the onset of symptoms is seen in around 20–30% of children [6,24].

The clinical spectrum of MRSA osteoarticular infections in children can range from uncomplicated infection with minimal tenderness or redness in the extremity to MRSA bacteremia and disseminated disease presenting with signs of sepsis, such as fever, chills, hemodynamic instability, tachycardia, and hypotension. As many as 50% of children with MRSA OSI have accompanying bacteremia [24]. MRSA infection of the bone can predispose to subperiosteal abscess formation and subsequent rupture into the soft tissues of the extremity may result in marked swelling and tenderness.

## 4. Complications

MRSA osteoarticular infections can be associated with severe purulent complications. In particular, Panton–Valentine leucocidin (PVL)-producing MRSA clones seem to be more aggressive with a higher predisposition for abscess formation (subperiosteal and intraosseous), the development of multiple infectious foci, and chronic osteomyelitis [24]. These strains have also been linked with thrombotic/embolic complications, including DVT (deep vein thrombosis), suppurative thrombophlebitis, and mainly pulmonary septic embolism [24]. Disseminated MRSA infections with sepsis can have high mortality. Several reports have shown that patients with OSI due to PVL-carrying *S. aureus* strains have a greater risk of developing pyomyositis, necrotizing fasciitis, and orthopedic sequelae, thus requiring more aggressive management with prolonged antimicrobials and multiple surgical debridement procedures [25,26,27,28]. Serious orthopedic complications in children include growth plate damage, limb shortening and limb length discrepancy, angular limb deformity, femoral head AVN (avascular necrosis), joint stiffness, and osteonecrosis [26].

## 5. Diagnosis

Initial laboratory testing must include a complete blood count (CBC) with differential, erythrocyte sedimentation rate (ESR), C-reactive protein (CRP), and a blood culture [6]. A blood culture is recommended due to the high prevalence of accompanying bacteremia and should be obtained before the initiation of antibiotics. The CBC is nonspecific and may show leukocytosis with marked neutrophilia, a left shift with a predominance of immature cells, or leucopenia with overwhelming sepsis. CRP and ESR are of value in dictating the duration of therapy as well as the transition of intravenous to oral antibiotic therapy [29]. Elevated ESR, white blood cell count, and CRP have been shown to have a sensitivity of 98% in diagnosing acute OSI [30]. Magnetic resonance imaging (MRI) with gadolinium is recommended as the imaging diagnostic method of choice, and is particularly useful in the diagnosis of early osteomyelitis [6,29]. MRI can show characteristic findings in osteomyelitis, such as cortical and marrow edema as well as the presence of soft tissue inflammatory changes/abscesses/fluid collections. Bone scans (usually with technetium-99) are sensitive in diagnosing osteoarticular infections and may be considered when suspecting OSI of an unknown anatomic site/multifocal disease and there is non-availability of MRI/pediatric anesthesia [6,31]. Ultrasound is of value in diagnosing septic arthritis of the hip [32]. Plain radiographs show the presence of a fracture but may not show acute changes until about 10 days into illness and hence are not reliable for the diagnosis of acute OSI [24].

If surgical intervention is undertaken, culture of the surgical/bone specimen or aspirated material is highly recommended. *S. aureus* on the Gram stain of the aspirated joint-fluid/surgical bone specimen appear as Gram-positive cocci in clusters. Once *S. aureus* is identified, laboratory methods to characterize MRSA are used.

## 6. Treatment of MRSA OSI

### 6.1. Antimicrobials

Early recognition and treatment of MRSA osteoarticular infections is essential for a good outcome. In a septic child, antibiotic therapy should be initiated at the earliest opportunity and not be delayed pending imaging or surgery [6,23]. Given the importance of MRSA as a pathogen in pediatric OSI, the empiric treatment of OSI must include an agent active against MRSA. The Infectious Diseases Society of America (IDSA) has formulated clinical practice guidelines for the management of MRSA infections [28].

According to the IDSA guidelines intravenous vancomycin is the antimicrobial agent of choice for acute hematogenous pediatric OSI due to MRSA [29]. Vancomycin is a glycopeptide antibiotic that inhibits the synthesis and assembly of cell wall peptidoglycan of bacteria [24]. Intravenous (IV) vancomycin is recommended at a dose of 15 mg/kg/dose every 6 h in order to maintain vancomycin serum through concentrations between 15 and 20 ug/mL for serious MRSA infections such as bacteremia and osteomyelitis [29]. Infections with *Staphylococcus aureus* isolates resistant to methicillin and intermediate to vancomycin [vancomycin intermediate staphylococcus aureus (VISA; vancomycin MIC 4–8 μg/mL)] or resistant to vancomycin [vancomycin resistant staphylococcus aureus (VRSA; vancomycin MIC > 16 μg/mL)] are associated with treatment failures with vancomycin, lack of clinical and microbiologic response and overall poor outcomes [29]. Although they are relatively uncommon, management of these infections is challenging.

Clindamycin (40 mg/kg/day) is an alternative antimicrobial for MRSA OSI in a stable patient without bacteremia or endovascular foci of infection, if clindamycin resistance rate is <10%, followed by oral therapy, given its high oral bioavailability and good bone penetration [29]. Clindamycin is a lincosamide antibiotic which acts by inhibition of bacterial protein synthesis. The main limitation in its use is resistance among MRSA isolates. In some recent studies, clindamycin resistance among MRSA isolates has been shown to range from 51 to 62% [33,34]. 

The *erm*(A) or *erm*(C) genes in the MRSA genome encode the methylase protein responsible for methylation of the 23S rRNA-binding site. Mutations in these genes confer inducible macrolide–lincosamide–streptogramin B (MLS_B_) resistance, resulting in resistance to clindamycin, macrolides and streptogramins (dalfopristin/quinopristin) [35,36]. In the microbiologic laboratory, the manifestation of this inducible MLS_B_ is characterized by the erythromycin-clindamycin D test, i.e., clindamycin resistance induction in the presence of erythromycin [6,36]. Moreover, *erm*(C) *S. aureus* isolates have been shown to have a much higher mean frequency of mutation to clindamycin resistance (14-fold) than that of the *erm*(A) *S. aureus* isolates [36]. Exposure to clindamycin of MLS_B_-inducible MRSA isolates may result in the selection of constitutive methylase-producing organisms as well as *erm* resistance. Therefore, the use of clindamycin clinically for patients with serious infections is discouraged in the presence of D-test-positive MRSA organisms. The other adverse effects of clindamycin include diarrhea and risk of *Clostridiodes (clostridium) difficile* colitis.

According to IDSA guidelines, daptomycin and linezolid are alternative antimicrobial agents for MRSA OSI. Daptomycin (6 mg/kg/day intravenously, once every 24 h) is a lipopeptide antibiotic that is bactericidal for MRSA. Although approved for MRSA BSI (loodstream infections), daptomycin is not useful for treating disseminated disease involving the lungs, due to its propensity for binding surfactant and consequent inactivation [24].

Linezolid is an oxazolidinone antibiotic that achieves good bone concentrations. It acts by binding to the bacterial 50S ribosomal subunit during formation of the initiation complex, thus inhibiting protein synthesis, and is bacteriostatic against MRSA [24]. The recommended dosage is 10 mg/kg/dose every 8 h for children less than 12 years of age and 600 mg oral/intravenous twice daily for children older than 12 years [29]. The limitations of linezolid are the cost and the frequent occurrence of neutropenia and thrombocytopenia with administration beyond 2 weeks. Resistance to linezolid occurs with prolonged use due to mutations in the 23SrRNA gene in the domain V region, of which *S. aureus* has five copies and mutations in two or more genes are needed for the resistance to manifest [24]. Newer antimicrobial agents active against MRSA include ceftaroline, an advanced-generation cephalosporin that binds to PBP2a, which has recently been approved for use in children (dosing range 24 mg/kg/day to 45 mg/kg/day every 8 h) hourly) [37,38]. It has been shown to have high efficacy in the treatment of skin and soft-tissue infections (SSTIs) in children [38]. Ceftobiprole is an advanced-generation parenteral cephalosporin active against MRSA that is similar to ceftaroline; however, ceftobiprole is approved in European countries but is not currently approved in the United States. Other antimicrobial agents approved for MRSA infections in adults include dalbavancin, oritavancin, and telavancin, which are glyco-lipopeptide derivatives like vancomycin [37]. Dalbavancin and oritavancin are approved for MRSA SSTIs in one or two doses 1–2 weeks apart, while telavancin is given once daily and is approved for adults with MRSA SSTIs and pneumonia. Tedizolid, which is a second-generation oxazolidinone like linezolid, is another agent recently approved for adults. It has been shown to be safe in adolescents; however, it is not presently approved for widespread use in pediatrics [39]. Daptomycin, linezolid and telavancin have been shown to be active against VISA and VRSA isolates [29]. 

### 6.2. Duration of Antimicrobial Treatment

In children with MRSA OSI, a total of 4–6 weeks of antibiotics for MRSA osteomyelitis and 3–4 weeks for septic arthritis are recommended [29].

The ideal duration of total and intravenous antibiotics still remains an area of active investigation and is yet to be determined, but clinical improvement and downward-trending CRP usually dictate the switch from intravenous antibiotics to oral antibiotics, and clinical resolution with normalization of ESR is relied on as a guide to adequate duration of therapy [6,20,24,40]. A study showed that switching to oral antibiotic therapy from intravenous when all subjective and objective criteria of improvement were met (CRP < 3, afebrile, and decreased pain) resulted in high cure rates [20]. Although another study showed that a short intravenous course (<5 days) followed by oral antibiotics to complete a total of 20–30 days was successful for treating most of the OSI by *S. aureus* (all Methicillin-sensitive *S. aureus*), none of the isolates were MRSA [41]. Short courses of antibiotics have not been evaluated in pediatric MRSA OSI, and further studies examining the successful use of shorter courses of IV and total antibiotic therapy in children with MRSA OSI are needed. The duration of intravenous vancomycin is more prolonged in patients with co-existing bacteremia, ranging between 2 and 6 weeks, depending on several factors, such as endovascular involvement, source, and metastatic infectious foci [29].

The general approach of transition from intravenous antibiotics to an oral antibiotic regimen should be directed by clinical response to treatment and must be individualized for each patient with MRSA OSI.

### 6.3. Surgical Intervention

Early surgical intervention is often required in OSI caused by MRSA, and the need for repeat interventions is usually indicated by the clinical response to treatment.

The IDSA guidelines state that prompt surgical debridement of soft tissue abscesses is essential and is recommended to be undertaken whenever feasible [29]. Surgical drainage is essential for clearing the source of the infection, especially in cases of persistent bacteremia or purulent collections in and around the bone [6,29]. The CRP is an important marker and studies have shown that a persistent undrained focus of purulence may be present if the CRP fails to decrease in the first 48 h or plateaus to a level more than 5 mg/dL [20,42]. In accordance with the treatment guidelines for septic arthritis in children, drainage or debridement of the joint space is recommended [29]. Surgical debridement is recommended specifically for the hips, while arthrocentesis may suffice for other septic joints.

## 7. Conclusions

In summary, MRSA is a major cause of pediatric morbidity and mortality in the United States and worldwide. Through evolution, MRSA has emerged as one of the predominant etiologic agents for pediatric OSI, and PVL-producing MRSA strains have been linked with severe disease manifestations. Prompt recognition and initiation of optimal antimicrobial treatment of MRSA OSI in children is vital for preventing disease dissemination as well as complications. In addition to appropriate antimicrobials, early surgical intervention is often necessary in MRSA OSI.
